# High blood eosinophils predict the risk of COPD exacerbation: A systematic review and meta-analysis

**DOI:** 10.1371/journal.pone.0302318

**Published:** 2024-10-03

**Authors:** Fangying Chen, Mei Yang, Hao Wang, Lian Liu, Yongchun Shen, Lei Chen

**Affiliations:** 1 Department of Pulmonary and Critical Care Medicine, West China Hospital, Sichuan University, Chengdu, Sichuan, China; 2 Department of Tuberculosis, The Third People’s Hospital of Tibet Autonomous Region, Lhasa, Tibet, China; 3 Laboratory of Pulmonary Diseases, West China Hospital, Sichuan University, Chengdu, Sichuan, China; Jhpiego Nigeria, NIGERIA

## Abstract

**Background:**

The association between blood eosinophils and COPD exacerbation has been controversial. This study aims to investigate whether high blood eosinophils predict the risk of COPD exacerbation across different thresholds and subgroups.

**Methods:**

PubMed, Embase and Web of science were searched for randomized controlled trial (RCT) and observational studies regarding the relationship between blood eosinophils and COPD exacerbation. Pooled risk ratio (RR) for COPD exacerbation was calculated using the Mantel-Haenszel method with a random-effects model.

**Results:**

A total of 21 studies (1 RCT and 20 observational studies) with 79868 participants were included. Thresholds of high blood eosinophils including absolute counts (200, 300 and 400 cell/μL) and percentages (2%, 3% and 4%) were analyzed respectively. Pooled analyses suggested that high blood eosinophils were significantly associated with increased risk of COPD exacerbation when using the thresholds of 300 cells/μL (RR 1.21, 95%CI 1.12–1.30, *P* <0.001, 16 studies), 400 cells/μL (RR 1.79, 95%CI 1.41–2.28, *P* <0.001, 3 studies), 2% (RR 1.26, 95%CI 1.02–1.55, *P* = 0.030, 10 studies) and 4% (RR 1.44, 95%CI 1.05–1.96, P = 0.022, 4 studies), but not 200 cells/μL and 3% (*P*>0.05). Moreover, high blood eosinophils contributed to moderate-severe exacerbation of COPD by the cutoffs of 300 cells/μL (RR 1.30, 95%CI 1.16–1.45, P<0.001, 11 studies) and 2% (RR 1.33, 95%CI 1.02–1.76, P = 0.037, 8 studies). In subgroup analyses, the pooled results further showed a significant association between high blood eosinophils (especially over 300 cells/μL) and risk of COPD exacerbation among patients from Europe and Asia, and whether with stable or exacerbation phase at baseline, and regardless of the follow-up time (≤ or > 1year).

**Conclusions:**

This study demonstrates that high blood eosinophils (over 300 cells/μL or 2%) could predict the risk of moderate-severe exacerbation of COPD in specific subgroups. However, large sample-sized, prospective, and well-designed studies are required to validate the present findings.

## Introduction

Chronic obstructive pulmonary disease (COPD) is a heterogeneous airway disease with significant morbidity and mortality [1]. According to the World Health Organization (WHO), COPD has been the third leading cause of death worldwide, putting a huge burden on global health-care systems [2]. Acute exacerbation of COPD (AECOPD) is the major cause of disease progression, increased hospitalization rate, prolonged hospital stay, poor quality of life and prognosis [3,4], contributing to the largest part of the total COPD burden [5]. The effective prediction and prevention of acute exacerbation has been key measures for COPD management, which depends on the investigation and controlling of risk factors. Currently, prediction for future risk of AECOPD mainly depends on the history of exacerbations in the prior year [6]. Specific and reliable indicators are still lacking.

Evidence has suggested that eosinophil plays a significant role in COPD. An unbiased cluster analysis revealed that 28% of acute exacerbation was associated with sputum eosinophilia, and the cut-off value of 2% blood eosinophils having a sensitivity of 90% and specificity of 60% for identifying sputum eosinophilia [7]. The 2024 Global Initiative for Chronic Obstructive Lung Disease (GOLD) guideline further underscores the importance of blood eosinophil count in guiding inhaled corticosteroids (ICS) therapy, and its association with airway pathogenic bacterial species [6]. Therefore, blood eosinophils could be potential biomarkers for predicting COPD progression and treatment effect.

Meanwhile, the relationship between blood eosinophils and risk of COPD exacerbation has attracted attention, but remains controversial [8–10]. During a three-year follow up, the COPDGene and ECLIPSE cohort studies indicated that high blood eosinophils (≥300 cells/μL) predicted future exacerbation outcome [9], while this finding was not repeated in some other reports [10,11]. Study on the CHAIN and BODE cohorts showed that blood eosinophils ≥300 cells/μL persisting over 2 years was not correlated with higher risk of AECOPD, although better survival was observed in this subgroup [10]. It is worth noting that, many previous studies included distinct subgroups, applied different thresholds, or failed to exclude patients with asthma, which cause potential confounding. Consequently, recent meta-analyses have examined the role of blood eosinophils in predicting the risk of acute exacerbation in COPD, but with uncertain conclusions [12,13]. Lately, it was reported AECOPD patients with blood EOS (≥ 2%) had higher readmission rates compared to those patients with no eosinophilia, indicating high blood EOS count could predict the risk of AECOPD [14].

To provide more comprehensive evidence, we systematically reviewed the current literature, to investigate whether high blood eosinophils predict increased risk of future COPD exacerbation. Given the cut-off value defining high blood eosinophils was undetermined, we analyzed the effects of different absolute (200, 300, 400 cells/μL) and relative (2%, 3%, 4%) thresholds respectively, which were widely used in previous studies or recommended by the guideline [11,15].

## Methods

### Searching strategy and selection criteria

This systematic review and meta-analysis was conducted according to the Preferred Reporting Items for Systematic Reviews and Meta-Analyses guideline (PRISMA 2020) [16]. PubMed, Embase and Web of Science were searched for reports regarding the relationship between blood eosinophils and AECOPD, from inception to October 01, 2023. Medical Subject Heading and Title/Abstract keywords were applied with the following search terms (using every possible combination): eosinophil, eosinophilia, chronic obstructive pulmonary disease and COPD. The search strategy was list in S1 Table. For the purpose of rapid review, the language was restricted to English [17]. Studies meeting the following criteria were included: 1) participants: adult patients (aged ≥18 years) with COPD, diagnosed according to the GOLD spirometry guideline (forced expiratory volume in 1 second (FEV_1_) / forced vital capacity(FVC)<0.7, post-bronchodilation) [6]; 2) exposure: providing data on baseline blood eosinophils of patients, and using at least one of the following cut-off values to define high blood eosinophils: 2%, 3%, 4%, 200 cells/μL, 300 cells/μL and 400 cells/μL; 3) outcome: exacerbation of COPD; 4) design: randomized controlled trials (RCTs) and observational studies (prospective or retrospective cohort studies, longitudinal studies). We excluded studies enrolling patients with asthma or other reported allergic diseases. Brief description of some excluded studies and the reasons for their exclusion were presented in S2 Table.

According to the selection criteria, two investigators (**FC and MY**) performed the initial search, independently screened titles and abstracts for studies and excluded duplicate or irrelevant records. For articles requiring further assessment, the full text was reviewed, and the references were manually checked to identify additional eligible studies. Disagreements were resolved by discussion between the two reviewers or with the help of the third investigator (**LC**).

### Data extraction and quality assessment

Two investigators (**FC** and **MY**) independently extracted data from each study using a standardized Excel file. The following information was picked up: first author (year of publication), country, study design, number of participants, demographic characteristics of patients, phases of COPD at baseline, maintenance treatment for COPD, thresholds of high blood eosinophils, follow-up time, definition of AECOPD and the outcome data. For the missing data, special effort was made to contact the corresponding authors of the original studies. Studies without available outcome data were not included in the quantitative analysis. The primary outcome was exacerbation of COPD. The secondary outcome was moderate-severe exacerbation. The quality of RCTs was evaluated with the Jadad Scale [18,19]. The 5-point Jadad Scale contains three items: randomization (0–2), blinding (0–2), and withdrawals/dropouts (0–1). Studies that scored over 3 points on the Jadad Scale were considered to be of high quality. The Newcastle-Ottawa Scale (NOS) was used to rate the risk of bias for observational studies [20], with a maximum score of 9 and a score of ≥6 indicating a low risk. Two investigators (**MY** and **HW**) independently assessed the quality of studies. Discrepancies were resolved through discussion with the third investigator (**LL**).

### Statistical analysis

Pooled effect estimates were expressed as RR with 95% confidence interval (CI). Statistical heterogeneity across studies was evaluated with the *I*^*2*^ statistic. *I*^*2*^ ≥ 50% indicated significant heterogeneity [21]. The Mantel-Haenszel method with a random-effects model was used to calculate pooled RRs and 95% CIs.

Because of undetermined cut-off value of high blood eosinophils currently, we chose to analyze the thresholds of 2%, 3%, 4%, 200, 300 and 400 cells/μL separately, which have been widely used and reported, especially 300 cells/μL and 2%. Moreover, based on the cut-offs of 300 cells/μL and 2%, subgroup analyses regarding region (Europe vs Asia vs North America vs Middle East), phase of COPD at baseline (stable vs exacerbation phase) and follow-up time (≤1 year vs >1 year) were conducted. We also performed the meta-regression to evaluate the effect of moderator variables (region, phase of COPD at baseline and follow-up time) on meta-analysis results. For sensitivity analysis, the influence of a single study on the overall pooled estimate was investigated by omitting one study in each turn. Publication bias was visually assessed with a funnel plot and evaluated by the Egger’s and Begg’s test [22]. *P* value <0.05 was considered statistically significant. Statistical analysis was performed using Stata 12.0 (Stata, College Station, TX, USA).

## Results

### Study selection

**Fig 1:**
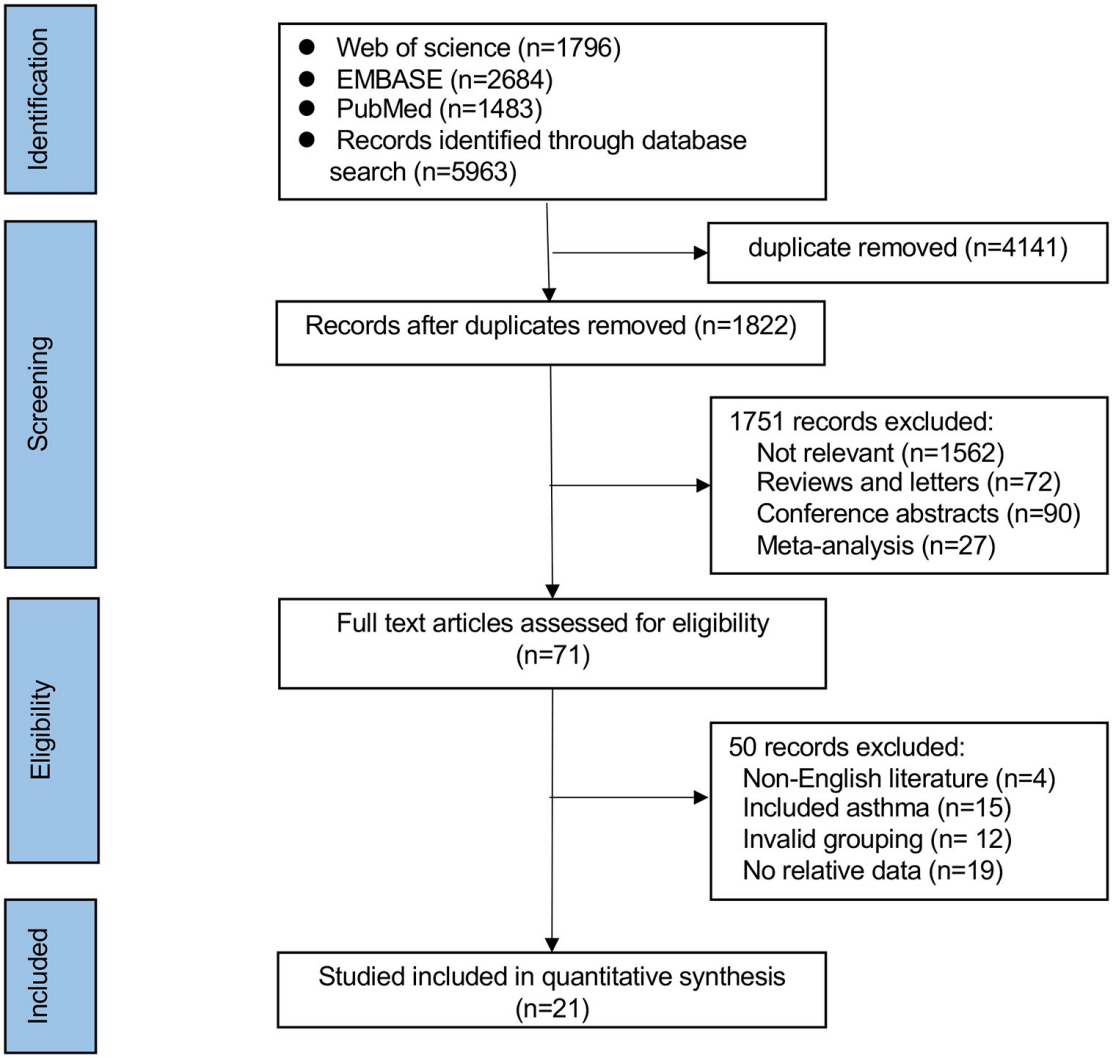
PRISMA flowchart outlining the literature search process.

The initial search yielded 5963 records. Based on the title and abstract, 4141 were removed for duplicates and 1751 were excluded for irrelevant data or ineligible study design (review, meta-analysis or letter). The remaining 71 full-text articles were screened for eligibility and 50 were excluded because of non-English literature (n = 4), included asthma (n = 15), invalid grouping (n = 12) or no relative data (n = 19). Finally, 21 articles were included in the meta-analysis [10,11,23–41]. The selection process is shown in Fig 1.

### Study characteristics and quality assessment

The characteristics of the 21 included studies are presented in Table 1. These studies were published between 2015 and 2022. The sample size ranged from 145 to 32693. Among these articles, 16 were cohort studies (13 retrospective [23–25,27,29–31,33–36,38,39], three prospective studies [10,37,41]), four were post-hoc analysis of RCTs [11,26,28,40] and only one was RCT [32]. Besides, 11 studies were conducted in Europe [10,11,25–28,32–36], six studies in Asia [29,37–41], two studies in the North America [24,31], and two studies in Middle East [23,30]. 15 studies [10,23–26,30,31,33–39,41] reported the outcome of moderate-severe exacerbation, defined as aggravation of respiratory symptoms that required treatment of systematic corticosteroids or/and antibiotics, hospitalization or visiting the emergency room. The remaining four studies did not specify the severity of exacerbation, which was defined as acute change of respiratory symptoms, warranting a change in therapy.

**Table 1:** Characteristics of the included studies.

Author/Year	Country	Study Design	NO. subjects (Male%)	Age (Years)	FEV1% pred	Treatment	Cut-off value	Follow-up	Outcome	NOS/Jadad
Adir (2018) [30]	Israel	Retrospective cohort	318 (65%)	71.4 ±11	49 ± 18%	Unknown	2%, 4% and 300 cells/μL	1 year	Exacerbation	6
Belanger (2018) [31]	Canada	Retrospective cohort	479 (52%)	68.9 ± 9.4	51.2 ± 16.8%	Corticosteroids, antimicrobial agents	2%, 3%, 4% and 200, 300, 400 cells/μL	1 year	Readmission	5
Bradbury (2022) [40]	China	Post-hoc analysis	1487 (76.5%)	64.6 ± 7.82	41.75 ± 15.12	theophylline, prednisone, steroids	2%, 3%, 4% and 200, 300, 400 cells/μL	1 year	Exacerbation	7
Casanova (2017) [10]	Spain	Prospective cohort	732 (85%)	67 ± 9	60 ± 19%	ICS/ Inhaled medication	300 cells/μL	2 years	Exacerbation	7
Chapman (2018) [32]	UK	RCT	1053 (70.6%)	65.3 ± 7.80	56.6 ± 9.97%	ICS/LABA/LAMA	2% and 300 cells/μL	26 weeks	Exacerbation	5
Duman (2015) [23]	Turkey	Retrospective cohort	1704 (66%)	70	≤70%	Unknown	2%	6 months	Exacerbation	7
Gonzalez-Barcala (2019) [34]	Spain	Retrospective cohort	1626 (77.1%)	67.34 ±11.1	49.4 ± 18.5%	Unknown	200, 300, 400 cells/μL	1 year	Readmission	5
Hakansson (2020) [35]	Denmark	Retrospective cohort	4022 (45%)	73.1 (63.7–81.1)	Unknown	Unknown	300 cells/μL	1 year	Readmission	7
Hasegawa (2016) [24]	USA	Retrospective cohort	3084 (50%)	70 (61–79)	Unknown	Unknown	300 cells/μL	1 year	Readmission	5
Jabarkhil (2020) [36]	Denmark	Retrospective cohort	811(41.3%)	73 (65–81)	40 (30–52) %	ICS/LABA/LAMA/SAMA/SABA	300 cells/μL	3 years	Exacerbation	8
Jo (2022) [41]	Korean	Prospective cohort	627 (93%)	68.8 ± 7.5	58.3 ± 18.2	ICS/LABA/LAMA	300 cells/μL	3 years	Exacerbation	6
Juthong (2020) [37]	Thailand	Prospective cohort	145 (94.5%)	73.9 ± 9.7	Unknown	ICS/LABA/LAMA	300 cells/μL	1 year	Readmission	7
Oshagbemi (2018) [33]	Netherlands	Retrospective cohort	32693 (55.5%)	68.4 ± 10.8	Unknown	Xanthine derivatives, Antipsychotics/SABA/LABA/LAMA/SAMA	2% and 4%	1 year	Exacerbation	7
Pavord (2016) [25]	UK	Retrospective cohort	1710 (79%)	64.1	≤ 70%	FP/SAL/Tiotropium/BD	2%	≥ 1 year	Exacerbation	5
Prins (2017) [27]	Netherlands	Retrospective cohort	207 (53%)	70	50.6 (16) %	Unknown	2% and 300 cells/μL	180 days	Exacerbation	5
Roche (2017) [28]	Netherlands, Belgium	Post-hoc analysis	3349 (73.1%)	64.2 (7.86)	44%	ICS/LABA/LAMA	2%, 3% and 300 cells/μL	1 year	Exacerbation	8
Singh (2020) [11]	UK	Post-hoc analysis	22125 (74.1%)	65.1 (8.6)	Unknown	ICS/LABA/LAMA	300cells/μL	1 year	Exacerbation	7
Song (2017) [29]	Korea	Retrospective cohort	467 (96%)	69.5 ± 7.4	55.5 ± 18%	ICS	200 cells/μL	1 year	Exacerbation	6
Watz (2016) [26]	UK	Post-hoc analysis	2096 (82%)	63.5	33.8%	ICS	2%, 3%, 4% and 300, 400 cells/μL	1 year	Exacerbation	9
Wu (2020) [38]	Taiwan	Retrospective cohort	625 (88%)	76.89±10.09	48.55 ± 17.69%	ICS/LABA/LAMA	2%	1 year	Exacerbation	5
Yu (2021) [39]	China	Retrospective cohort	508 (72.0%)	76.5 ± 8.8	52.05 ± 19.20%	Unknown	300 cells/μL	1 year	Exacerbation	7
**Total 21 articles**			**Patients 79868**									

LABA, Long-acting β-agonist; LAMA, Long-acting Muscarinic Antagonist; SABA, Short-Acting Beta Agonist; SAMA, Short-Acting Muscarinic Antagonist; BD, bronchodilator; FP, fluticasone propionate; SAL, salmeterol; NOS, Newcastle-Ottawa Scale; Jadad, Jadad scale.

A total of 79868 participants were included in this meta-analysis. The mean age ranged from 63 to 77 years. The proportion of male subjects was between 41% and 96%. The mean FEV_1_% predicted of patients was above 30%, and more than 44.7% of them were receiving ICS-containing therapies.

Quality assessment of included studies are presented in S3 Table. The RCT described the generation of a randomized sequence, a double-blind design, and reported the numbers and reasons of withdrawal or dropout in detail, indicating a low risk of bias [32]. According to the NOS, 14 observational studies were considered at low risk of bias (scored ≥6) and 6 studies [24,25,31,32,34,38] were rated a score of 5.

### Association between high blood eosinophils and risk of AECOPD according to different thresholds

**Fig 2:**
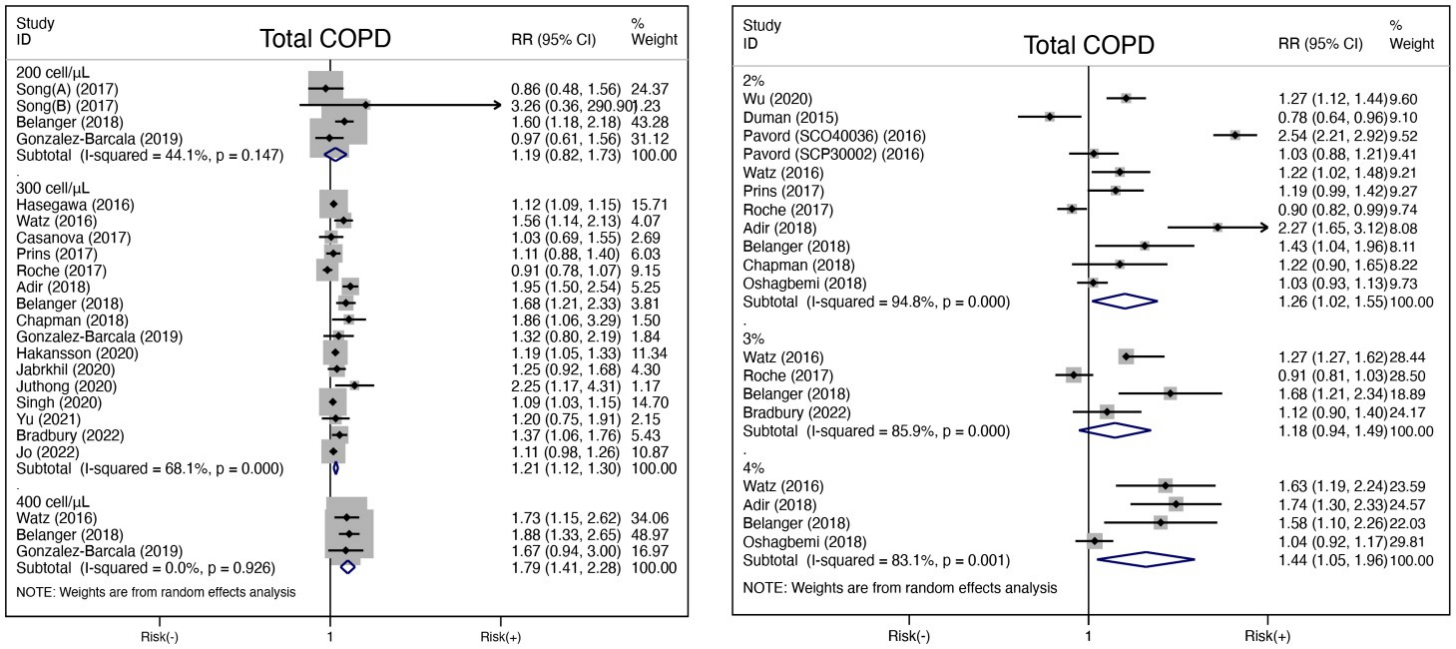
Risk of COPD exacerbation in relation to high blood eosinophils, based on different thresholds. (A) High blood eosinophil was defined using the thresholds of 200, 300 and 400 cells/μL respectively. (B) High blood eosinophil was defined using the thresholds of 2%, 3% and 4% respectively. *(Note*: *Song(A)*, *participants using ICS/LABA during the study; Song(B)*, *participants not using ICS/LABA during the study*. *Pavord (SCO40036)*, *participants from the RCT SCO40036; Pavord (SCP30002)*, *participants from the RCT SCP30002)*.

A total of 17 studies [10,11,24,26–32,34–36,39–42] applied thresholds of absolute counts (200, 300 and 400 cells/μL) to define high blood eosinophils. As shown in Fig 2A, pooled analysis showed that high blood eosinophils were significantly associated with increased risk of future COPD exacerbation when using the thresholds of 300 cells/μL (RR 1.21, 95%CI 1.12–1.30, *P* <0.001, *I*^*2*^ = 68.1, 16 studies) and 400 cells/μL (RR 1.79, 95%CI 1.41–2.28, *P* <0.001, *I*^*2*^ = 0%, 3 studies). However, no significant association was observed when applying the cut-off of 200 cells/μL (RR 1.19, 95%CI 0.82–1.73, *P* = 0.366, *I*^*2*^ = 44.1%, 3 studies).

Besides, 11 studies [23,25–28,30–33,38,40] reported data on thresholds of percentages (2%, 3% and 4%). As shown in Fig 2B, pooled analysis revealed high blood eosinophils statistically significantly correlated with increased risk of AECOPD when applying the thresholds of 2% (RR 1.26, 95%CI 1.02–1.55, *P* = 0.030, *I*^*2*^ = 94.8%, 10 studies) and 4% (RR 1.44, 95%CI 1.05–1.96, *P* = 0.022, *I*^*2*^ = 83.1%, 4 studies), but not 3% (RR 1.18, 95%CI 0.94–1.49, *P* = 0.152, *I*^*2*^ = 85.9%, 4 studies), with apparent heterogeneity across studies.

**Fig 3:**
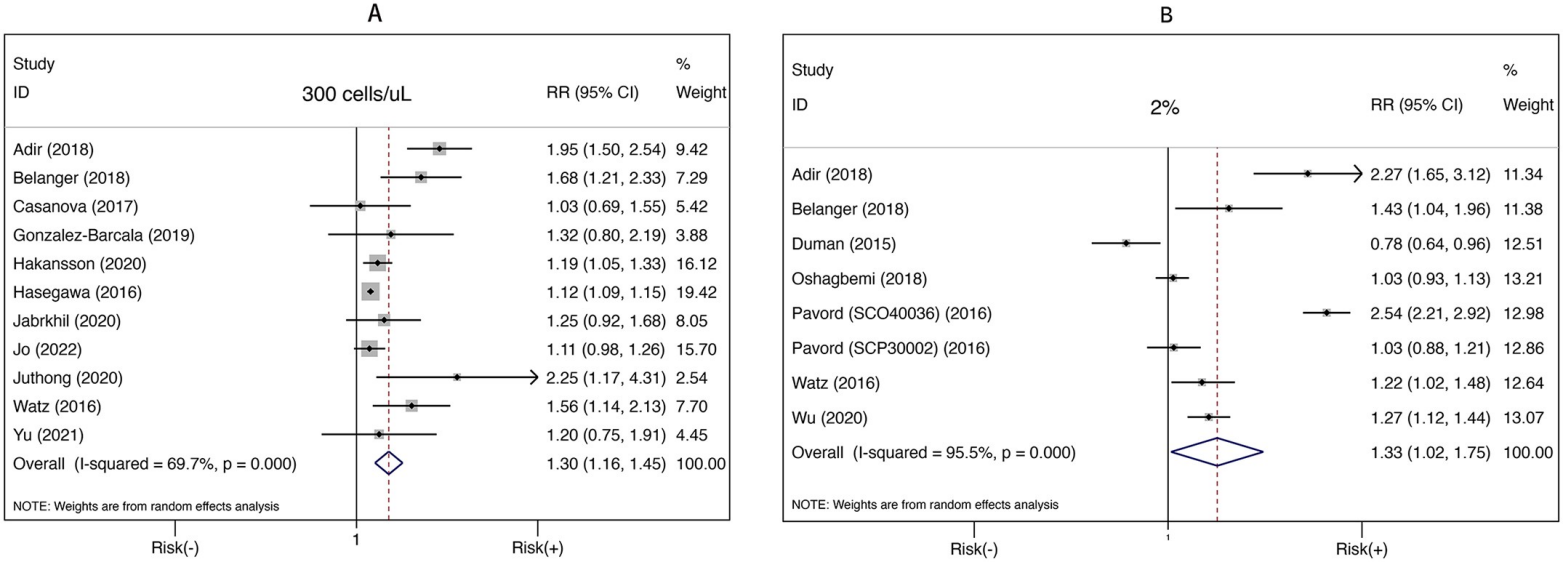
Risk of moderate-severe exacerbation in relation to high blood eosinophils, based on the thresholds of 300 cells/μL and 2%. (A) High blood eosinophil was defined as ≥300 cells/μL. (B) High blood eosinophil was defined as ≥ 2%.

Among studies reporting the outcome of moderate-severe exacerbation, the majority applied the thresholds of 300 cells/μL [10,24,26,30,31,34–37,41] and 2% [23,25,26,30,31,33,38]. As shown in Fig 3, elevated blood eosinophils were markedly correlated with increased risk of moderate-severe exacerbation when using 300 cells/μL (RR 1.30, 95%CI 1.16–1.45, *P* <0.001, *I*^*2*^ = 69.7%, 11 studies) and 2% (RR 1.33, 95%CI 1.02–1.75, *P* = 0.037, *I*^*2*^ = 95.5%, 7 studies) respectively, but also with apparent heterogeneity.

### Subgroup analyses and meta-regression

Subgroup analyses using the thresholds of 300 cells/μL and 2% were shown in Table 2 and S1–S3 Figs. As results, the association between high blood eosinophils and risk of COPD exacerbation were inconsistent across different regions and follow-up time. Results on the threshold of 300 cells/μL appeared more stable than the 2%, which revealed that the association between high blood eosinophils and exacerbation risk was statistically significant in participants from Asia (RR 1.13, 95%CI 1.10–1.15, *P* = 0.025, *I*^*2*^ = 49.7%, 4 studies), Europe (RR 1.14, 95%CI 1.03–1.26, *P* = 0.009, *I*^*2*^ = 51.8%, 9 studies) or Middle East (RR 1.95, 95%CI 1.50–2.54, *P* <0.001, 1 study) (S1 Fig), whether with stable (RR 1.49, 95%CI 1.07–2.09, *P* = 0.018, *I*^*2*^ = 80.5%, 5 studies) or exacerbation phase (RR 1.15, 95%CI 1.09–1.20, *P* <0.001, *I*^*2*^ = 8.8%, 8 studies) at baseline (S2 Fig), or the follow-up time less (RR 1.23, 95%CI 1.13–1.34, *P* <0.001, *I*^*2*^ = 74.1%, 13 studies) or more than (RR 1.12, 95%CI 1.00–1.25, *P* = 0.043, *I*^*2*^ = 0.0%, 3 studies)1 year (S3 Fig). However, significant heterogeneity was also observed and the meta-regression suggested that region, phase of COPD at baseline and follow-up time did not markedly contributed to the heterogeneity (*P*>0.05) (S4 Table).

**Table 2:** Results of subgroup analyses.

Subgroups	No. Studies	RR (95% CI)	P value	*I*^*2*^, %
**Region**				
***Middle East***				
300cells/μL	1	1.95[1.50–2.54]	*P <0*.*001*	--
2%	2	1.32[0.47–3.76]	*P = 0*.*600*	96.8%
***N*. *America***				
300cells/μL	2	1.33[0.90–1.97]	*P = 0*.*159*	83.2%
2%	1	1.43[1.04–1.96]	*P = 0*.*026*	--
***Europe***				
300cells/μL	9	1.14[1.03–1.26]	*P = 0*.*009*	51.8%
2%	7	1.23[0.93–1.62]	*P = 0*.*140*	96.2%
***Asia***				
300cells/μL	4	1.13[1.10–1.15]	*P = 0*.*025*	49.7%
2%	1	1.27[1.03–1.57]	*P <0*.*001*	--
**Phases of COPD at baseline**				
***Acute exacerbation***				
300cells/μL	8	1.15[1.09, 1.20]	*P <0*.*001*	8.8%
2%	5	1.07[0.86, 1.33]	*P = 0*.*527*	82.2%
***Stable phase***				
300 cells/μL	5	1.49[1.07, 2.09]	*P = 0*.*018*	80.5%
2%	5	1.49[0.97, 2.28]	*P = 0*.*067*	96.9%
**Follow-up time**				
***≤ 1 year***				
300cells/μL	13	1.23[1.13–1.34]	*P <0*.*001*	74.1%
2%	9	1.17[1.01–1.36]	*P = 0*.*042*	86.4%
***> 1 year***				
300cells/μL	3	1.12[1.00–1.25]	*P = 0*.*043*	0.0%
2%	2	1.62 [0.67–3.91]	*P = 0*.*282*	98.6%

(Note: Weights are from random effects analysis).

### Sensitivity analysis and publication bias

Further exclusion of any single study did not materially alter the pooled RRs, whether using the cutoff of 300 cells/μL or 2% (S4 Fig). Based on the funnel plot and statistical tests, no significant publication bias was found with the cutoff of 300 cells/μL(Egger’s test, p = 0.099; Begg’s test, *p = 0*.*115*) (S5 Fig).

## Discussion

This study conducted a comprehensive investigation to examine the association between high blood eosinophils and the risk of exacerbation in patients with COPD. The findings revealed that this association varied across different thresholds (200, 300, 400 cells/μL and 2%, 3%, 4%) and factors (including different regions, phases of COPD, and follow-up time). However, it was observed that high blood eosinophils (exceeding 300 cells/μL and 2%) may potentially predict the risk of moderate-to-severe exacerbation of COPD in specific subgroups.

Considering the burden of AECOPD, effective prevention strategies are urgently required. Further interpretation on the predictors or risk factors of AECOPD could optimize the disease prevention and control. Currently, although the number of self-reported previous exacerbation could help predict future risk, it is ineffective for most patients with uncertain history of exacerbation, for whom a specific biomarker is always needed. The potential of eosinophils has been suggested. As a part of leukocytes, eosinophil plays a prominent role in immune homeostasis and inflammatory diseases [42]. Zhu et al. revealed that Cysteinyl leukotriene 1 receptor protein, whose ligand was released by eosinophils, was apparently greater in COPD patients with severe exacerbation than those in stable phase [43]. The gene expression metric of Th2-high asthma was also elevated in patients with COPD, and further associated with decreased lung function, increased airway and blood eosinophils [44]. The eosinophil-associated phenotype of AECOPD was also indicated [7]. These suggested that eosinophil could play an important part in the development and progression of COPD. There is a potential link between elevated blood eosinophils and exacerbation of COPD. Exacerbations of chronic bronchitis are often accompanied by significant airway eosinophilia [45], characterized by the release of inflammatory mediators including interleukin-4 (IL-4), interleukin-5 (IL-5), and interleukin-13 (IL-13). These mediators can induce airway inflammation and remodeling, contributing to the deterioration of COPD [46]. Besides, blood eosinophils have been suggested as biomarkers for guiding ICS therapy. The debated association between high blood eosinophils and risk of COPD exacerbation has been reported, as cut-off value defining high blood eosinophils always controversial. The cut-off value could depend on the median count of blood eosinophils in patients with COPD, which also varied across subgroups [47]. Using different thresholds, we found that results on the 300 cells/μL appeared more stable than other thresholds and 300 cells/μL of blood eosinophil count was also the cut-off value recommended by the GOLD guideline when considering ICS therapy. In the IMPACT analysis, the magnitude of benefit of ICS-containing treatment in reducing moderate-severe exacerbation significantly increased when blood eosinophil count more than 300 cells/μL [48]. These findings may underscore potential value of this threshold in AECOPD.

Based on the threshold of 300 cells/μL, we found a 1.21 folds increase in the risk ratios of exacerbation and a 1.30 folds increase in risk of moderate-severe exacerbation in patients with high blood eosinophils. It is well known that moderate-severe exacerbation is frequently associated with bacterial infection. A recent study indicates that blood eosinophils count is positively linked with the percent of Firmicutes and streptococcus, which are the common pathogens of AECOPD [49]. The multi-omics analyses further proposed the airway host-microbe interactions achieved through inflammatory cells, including eosinophils [50]. These interactions may play key roles in the development of COPD. Meanwhile, when using the threshold of 300 cells/μL in subgroup analyses, similar mild association was revealed in participants from Asia or Europe, with stable or exacerbation phase, or with follow-up time less than one year. No significant correlation was observed in patients from North America or with follow-up time more than one year. These findings seem to be consistent with previous evidence. Blood eosinophil count has been reported to be higher in patients from North America than Asia. The NHANES 2007–2008 and NHANES 2009–2010 study revealed a median blood eosinophil count of 200 cell/μL [51], while the Nagahama study indicated a median count of 120 cells/μL among Japanese participants [52]. This might explain the opposite results between patients from Asia and North America in the present study. Additionally, the reproducibility of blood eosinophils ≥300 cells/μL in two years was reported lower than 20%, contrasting with the good reproducibility within one year [10], suggesting a good short-term stability of this cut-off value, which might explain the opposite findings between follow-up times (≤1 year vs >1 year). However, given the significant heterogeneity across studies and population-related variance of blood eosinophil counts, the mild association revealed in the present study needs to be interpreted with caution.

Studies on blood eosinophils and AECOPD have presented significant heterogeneity. Using the meta-regression, we found that different regions, phases of COPD at baseline and follow-up time were not the main sources of variance. Instead, we infer different thresholds of high blood eosinophils could contribute to the heterogeneity, based on the controversial results on these thresholds. Therefore, the larger and well-designed studies with distinct subgroups categorized by different thresholds are warranted.

This study has several limitations. First, only one RCT was included while others were observational studies. The limitations of observational study design may weaken the reliability of study results. Second, significant heterogeneity observed across studies and the limited number of studies in subgroup analyses may affect the robustness of study results. Third, given the English-language restriction in study searching, studies in non-English languages with negative results were not included. Thus, publication bias may exist and also weaken the robustness of our findings, although the Egger’s and Begg’s test revealed no significant bias. Fourth, other factors could influence the relationship between blood eosinophils and risk of AECOPD, such as smoking, history of exacerbations and ICS, which we failed to analyze because of limited data. To promote a better interpretation regarding this relationship, adjusting confounding by these factors is particularly important in future studies.

In conclusion, this study suggests that high blood eosinophil counts, particularly over 300 cells/μL or 2%, could serve as a prognostic indicator for moderate-to-severe exacerbations of COPD in specific subgroups. However, large sample-sized, prospective, and well-designed studies are required to validate the present findings.

## Supporting information

S1 Table Literature online search strategies. (DOCX)

S2 Table Brief description of some excluded studies and the reasons for their exclusion. (DOCX)

S3 Table Quality assessment of the included studies. (DOCX)

S4 Table Results of regression analysis with 2% and 300 cells /μL threshold. (DOCX)

S1 Fig Risk of COPD exacerbation in relation to high blood eosinophils across different regions, based on the thresholds of 300 cells/μL and 2%. (A) High blood eosinophil was defined as ≥300 cells/μL. (B) High blood eosinophil was defined as ≥2%.
(DOCX)

S2 Fig Risk of COPD exacerbation in relation to high blood eosinophils in different phases of COPD at baseline, based on the thresholds of 300 cells/μL and 2%. (A) High blood eosinophil was defined as ≥300 cells/μL. (B) High blood eosinophil was defined as ≥2%. (*Note*: *The study of Adir (2018) included two subgroups*, *with AECOPD and stable COPD at baseline respectively)*.
(DOCX)

S3 Fig Risk of COPD exacerbation in relation to high blood eosinophils with different follow-up time, based on the thresholds of 300 cells/μL and 2%. (A) High blood eosinophil was defined as ≥300 cells/μL. (B) High blood eosinophil was defined as ≥2%.
(DOCX)

S4 Fig Sensitivity analysis, based on the thresholds of 300 cells/μL (A) and 2% (B). Further exclusion of any single study did not materially alter the pooled RRs.
(DOCX)

S5 Fig Publication bias, based on the threshold of 300 cells/μL. (A) Funnel plot. (B) Egger’s publication bias plot.
(DOCX)

## Abbreviations

AECOPD: Acute exacerbation of COPD
CI: confidence interval
COPD: chronic obstructive pulmonary disease
FEV_1_: forced expiratory volume in 1 second
FVC: forced vital capacity
GOLD: Global Initiative for Chronic Obstructive Lung Disease
ICS: inhaled corticosteroids
Jadad: Jadad scale
NOS: Newcastle-Ottawa Scale
PRISMA: Preferred Reporting Items for Systematic Reviews and Meta-Analyses
RCT: randomized controlled trial
RR: risk ratio
WHO: World Health Organization
