# Supplementary material for: High blood eosinophils predict the risk of COPD exacerbation: A systematic review and meta-analysis
Source: PLoS One. 2024 Oct 3;19(10):e0302318. doi: 10.1371/journal.pone.0302318 (PMC11449345; doi:10.1371/journal.pone.0302318)
Supplement: S1 Table — (DOCX) [file pone.0302318.s001.docx]

**S1 Table.** **Literature online search strategies.**

| **Database** | **Search strategy** |
| --- | --- |
| PubMed | ((eosinophilia) [MeSH Terms] OR (eosinophil) [MeSH Terms]  OR (eosinophilia) [All Fields] OR (eosinophil) [All Fields]) AND  ((chronic obstructive pulmonary disease) [Title/Abstract] OR (chronic obstructive pulmonary disease) [MeSH Terms] OR (COPD)[Title/Abstract]) |
| Embase | ('eosinophilia'/exp OR 'eosinophil'/exp) AND  ('COPD'/exp OR 'chronic obstructive pulmonary disease'/exp) |
| Web of Science | (ALL = ‘eosinophilia’ OR ALL=‘eosinophil’) AND  (ALL = ‘COPD’ OR ALL=‘chronic obstructive pulmonary disease’) |
