# Supplementary material for: High blood eosinophils predict the risk of COPD exacerbation: A systematic review and meta-analysis
Source: PLoS One. 2024 Oct 3;19(10):e0302318. doi: 10.1371/journal.pone.0302318 (PMC11449345; doi:10.1371/journal.pone.0302318)
Supplement: S2 Table — (DOCX) [file pone.0302318.s002.docx]

**S2 Table. Brief description of some excluded studies and the reasons for their exclusion.**

| **50 records excluded** |
| --- |
| **Non-English literature (n=4)** |
| (Bedolla-Barajas, et al. 2021; Jiao, et al. 2020; Saad, et al. 2019; Zhang and Lin 2019) |
|  |
| **Included asthma (n=15)** |
| (Chan, et al. 2020; Hastie, et al. 2017; Hegewald, et al. 2020; Kang, et al. 2021; Landis, et al. 2018; Martinez-Gestoso, et al. 2021; Miravitlles, et al. 2021; Negewo, et al. 2016; Nishimura, et al. 2021; Pascoe, et al. 2015; Vedel-Krogh, et al. 2016; Xu, et al. 2022; Yun, et al. 2018; Zeiger, et al. 2018; Zysman, et al. 2017) |
|  |
| **Invalid grouping (n= 12)** |
| (Couillard, et al. 2017; Csoma, et al. 2021; Ferguson, et al. 2018; Hinds, et al. 2016; Jiang, et al. 2020; Kerkhof, et al. 2020b; Kostikas, et al. 2021; Mullerova, et al. 2019; Peng, et al. 2021; Siddiqui, et al. 2015; Vedel-Krogh, et al. 2018; Zhang, et al. 2020) |
|  |
| **No relative data (n=19)** |
| (Aksoy, et al. 2018; Bafadhel, et al. 2016; Bafadhel, et al. 2012; Barnes, et al. 2016; Cheng and Lin 2016; Disantostefano, et al. 2016; Kerkhof, et al. 2020a; Kerkhof, et al. 2017; Kim, et al. 2017; Li, et al. 2019; Lv, et al. 2021; Mendy, et al. 2018; Nishimura, et al. 2021; Oshagbemi ; Oshagbemi, et al. 2019; Papi, et al. 2017; Serafino-Agrusa, et al. 2016; Vestbo, et al. 2017; Xu, et al. 2022) |
