# Supplementary material for: High blood eosinophils predict the risk of COPD exacerbation: A systematic review and meta-analysis
Source: PLoS One. 2024 Oct 3;19(10):e0302318. doi: 10.1371/journal.pone.0302318 (PMC11449345; doi:10.1371/journal.pone.0302318)
Supplement: S3 Table — (DOCX) [file pone.0302318.s003.docx]

**S3 Table. Quality assessment of the included studies.**

| **Study/Year** | **Study design** | **Selection**  ******** | **Comparability**  ****** | **Outcome / Exposure ***** | **NOS** |
| --- | --- | --- | --- | --- | --- |
|  |  | **Randomization**  ****** | **Blinding**  ****** | **Withdrawals /Dropouts *** | **Jadad** |
| Adir (2018) | Retrospective cohort | ** | ** | ** | 6 |
| Belanger (2018) | Retrospective cohort | *** | * | * | 5 |
| Bradbury (2022) | Post-hoc analysis | ** | ** | *** | 7 |
| Casanova (2017) | Longitudinal | *** | ** | ** | 7 |
| Chapman (2018) | RCT | ** | ** | * | 5 |
| Duman (2015) | Retrospective cohort | *** | ** | ** | 7 |
| Gonzalez-Barcala (2019) | Retrospective cohort | *** | * | * | 5 |
| Hakansson (2020) | Retrospective cohort | *** | ** | ** | 7 |
| Hasegawa (2016) | Retrospective cohort | *** | * | * | 5 |
| Jabrkhil (2020) | Retrospective cohort | **** | ** | ** | 8 |
| Jo (2022) | Prospective cohort | *** | * | ** | 6 |
| Juthong (2020) | Prospective cohort | *** | ** | ** | 7 |
| Oshagbemi (2018) | Retrospective cohort | *** | ** | ** | 7 |
| Pavord (2016) | Retrospective cohort | ** | ** | * | 5 |
| Prins (2017) | Retrospective cohort | ** | * | ** | 5 |
| Roche (2017) | Post-hoc analysis | *** | ** | *** | 8 |
| Singh (2020) | Post-hoc analysis | ** | ** | *** | 7 |
| Song (2017) | Retrospective cohort | *** | ** | * | 6 |
| Watz (2016) | Post-hoc analysis | **** | ** | *** | 9 |
| Wu (2020) | Retrospective cohort | ** | * | ** | 5 |
| Yu (2021) | Retrospective cohort | *** | ** | ** | 7 |

**RCT**, based on Jadad scale, randomization (2 stars), blinding (2 stars), and withdrawals/dropouts (1 stars).

**Observational Studies**, based on the Newcastle-Ottawa Scale, maximum number of stars assigned for each category: Selection (4 stars), Comparability (2 stars), Outcome (3 stars).
