# Supplementary material for: High blood eosinophils predict the risk of COPD exacerbation: A systematic review and meta-analysis
Source: PLoS One. 2024 Oct 3;19(10):e0302318. doi: 10.1371/journal.pone.0302318 (PMC11449345; doi:10.1371/journal.pone.0302318)
Supplement: S4 Table — (DOCX) [file pone.0302318.s004.docx]

**S4 Table. Results of regression analysis with 2% and 300 cells /μL threshold**

| . metareg lnRR Region, COPD Stage, Follow-up, Exacerbation, wsse(selnRR) eform | | | | | | |
| --- | --- | --- | --- | --- | --- | --- |
| Meta-regression | | | | Number of obs | = | 11 |
| REML estimate of between-study variance | | | | tau2 | = | 0.1212 |
| % residual variation due to heterogeneity | | | | I-squared_res | = | 85.18% |
| Proportion of between-study variance explained | | | | Adj R-squared | = | -55.29% |
| Joint test for all covariates | | | | Model F(4,6) | = | 0.38 |
| With Knapp-Hartung modification | | | | Prob> F | = | 0.8138 |
| **lnRR of 2%** | exp(b) | Std. Err. | t | P>\|t\| | [95% Conf. Interval] | |
| Region | 1.031977 | 0.1517985 | 0.21 | 0.838 | 0.7200374 | 1.479057 |
| COPD Stage | 0.8979441 | 0.1607902 | -0.6 | 0.57 | 0.5793762 | 1.391676 |
| Follow-up | 1.17529 | 0.4287542 | 0.44 | 0.673 | 0.4813618 | 2.869581 |
| Exacerbation | 0.9173515 | 0.2586907 | -0.31 | 0.77 | 0.4601112 | 1.828979 |
| _cons | 1.243519 | 0.9512038 | 0.28 | 0.785 | 0.1913274 | 8.082162 |

| . Metareg lnRR Region, COPD Stage, Follow-up, Exacerbation, wsse(selnRR) eform graph | | | | | | |
| --- | --- | --- | --- | --- | --- | --- |
| Meta-regression | | | | Number of obs | = | 16 |
| REML estimate of between-study variance | | | | tau2 | = | 0.01126 |
| % residual variation due to heterogeneity | | | | I-squared_res | = | 34.71% |
| Proportion of between-study variance explained | | | | Adj R-squared | = | .% |
| Joint test for all covariates | | | | Model F(4,11) | = | 0.72 |
| With Knapp-Hartung modification | | | | Prob> F | = | 0.5981 |
| **lnRR of 300 cells /μL** | exp(b) | Std. Err. | t | P>1t\| | [95% Conf. Interval] | |
| Region | 1.011051 | 0.0855211 | 0.13 | 0.899 | 0.8393034 | 1.217943 |
| COPD Stage | 0.9302688 | 0.1039289 | -0.65 | 0.531 | 0.7274762 | 1.189592 |
| Follow-up | 0.8389687 | 0.1568157 | -0.94 | 0.368 | 0.5560047 | 1.26594 |
| Exacerbation | 0.9028972 | 0.1265581 | -0.73 | 0.481 | 0.6632149 | 1.229199 |
| _cons | 1.853913 | 0.6090136 | 1.88 | 0.087 | 0.8996687 | 3.820288 |
