# Supplementary material for: High blood eosinophils predict the risk of COPD exacerbation: A systematic review and meta-analysis
Source: PLoS One. 2024 Oct 3;19(10):e0302318. doi: 10.1371/journal.pone.0302318 (PMC11449345; doi:10.1371/journal.pone.0302318)
Supplement: S2 Fig — (A) High blood eosinophil was defined as ≥300 cells/μL. (B) High blood eosinophil was defined as ≥2%. (Note: The study of Adir (2018) included two subgroups, with AECOPD and stable COPD at baseline respectively). (DOCX) [file pone.0302318.s006.docx]

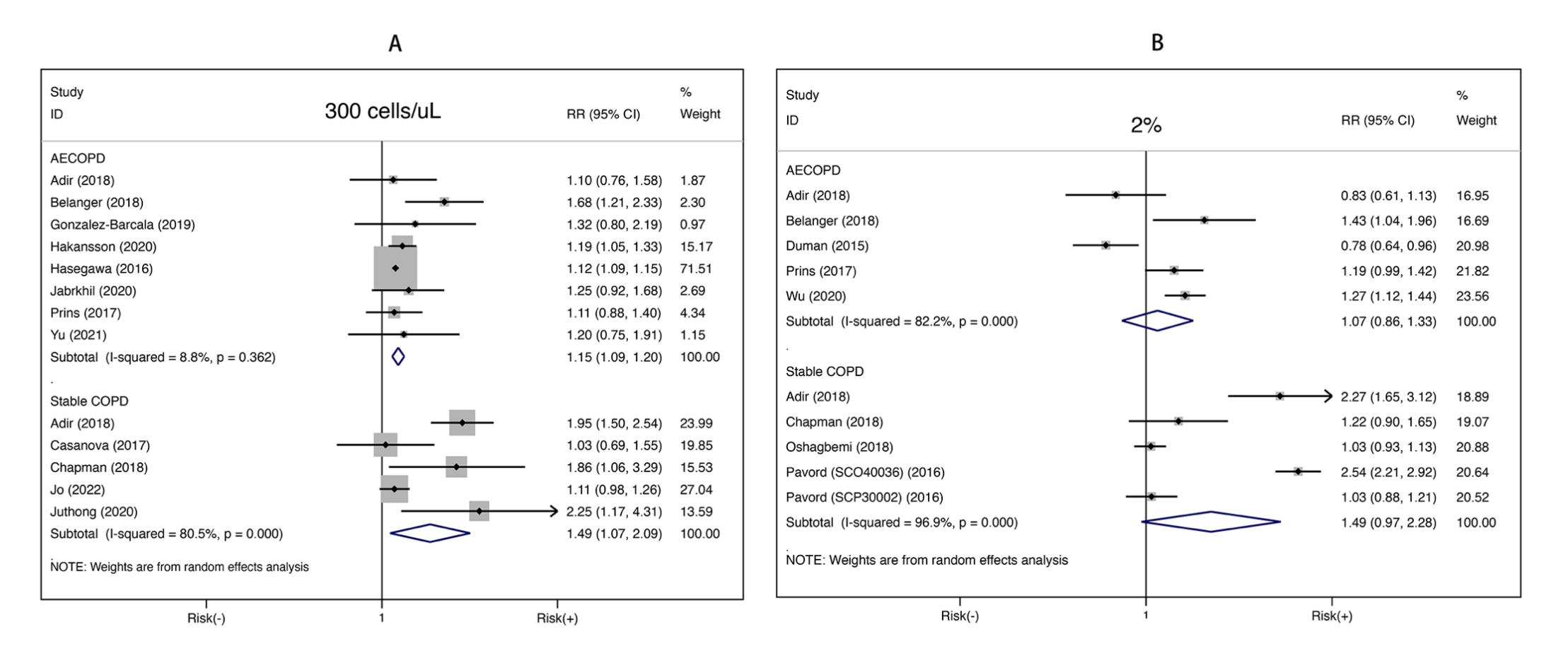
**S2 Fig.** Risk of COPD exacerbation in relation to high blood eosinophils in different phases of COPD at baseline, based on the thresholds of 300 cells/μL and 2%. (A) High blood eosinophil was defined as ≥300 cells/μL. (B) High blood eosinophil was defined as ≥2%. *The study of Adir (2018) included two subgroups, with AECOPD and stable COPD at baseline respectively.*
