# Supplementary material for: High blood eosinophils predict the risk of COPD exacerbation: A systematic review and meta-analysis
Source: PLoS One. 2024 Oct 3;19(10):e0302318. doi: 10.1371/journal.pone.0302318 (PMC11449345; doi:10.1371/journal.pone.0302318)
Supplement: S3 Fig — (A) High blood eosinophil was defined as ≥300 cells/μL. (B) High blood eosinophil was defined as ≥2%. (DOCX) [file pone.0302318.s007.docx]

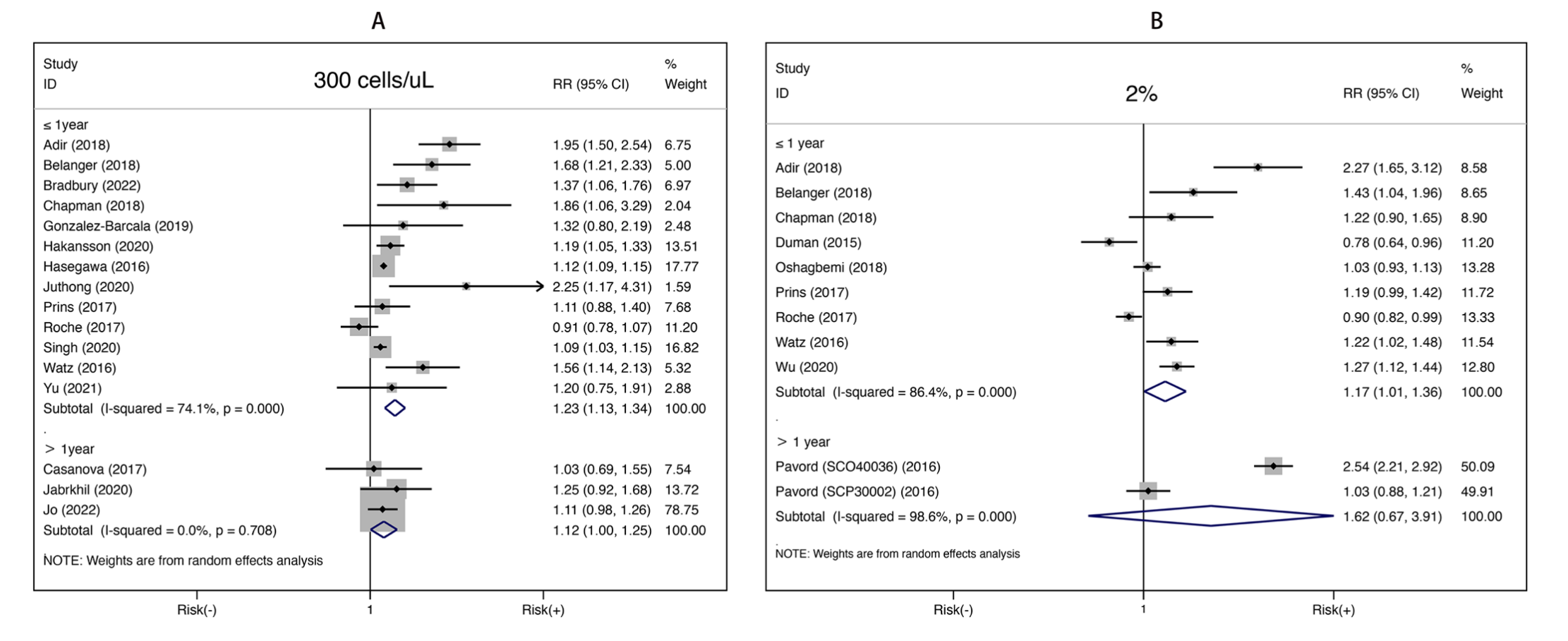
**S3 Fig.** Risk of COPD exacerbation in relation to high blood eosinophils with different follow-up time, based on the thresholds of 300 cells/μL and 2%. (A) High blood eosinophils was defined as ≥300 cells/μL. (B) High blood eosinophils was defined as ≥2%.
