# Supplementary material for: High blood eosinophils predict the risk of COPD exacerbation: A systematic review and meta-analysis
Source: PLoS One. 2024 Oct 3;19(10):e0302318. doi: 10.1371/journal.pone.0302318 (PMC11449345; doi:10.1371/journal.pone.0302318)
Supplement: S4 Fig — Further exclusion of any single study did not materially alter the pooled RRs. (DOCX) [file pone.0302318.s008.docx]

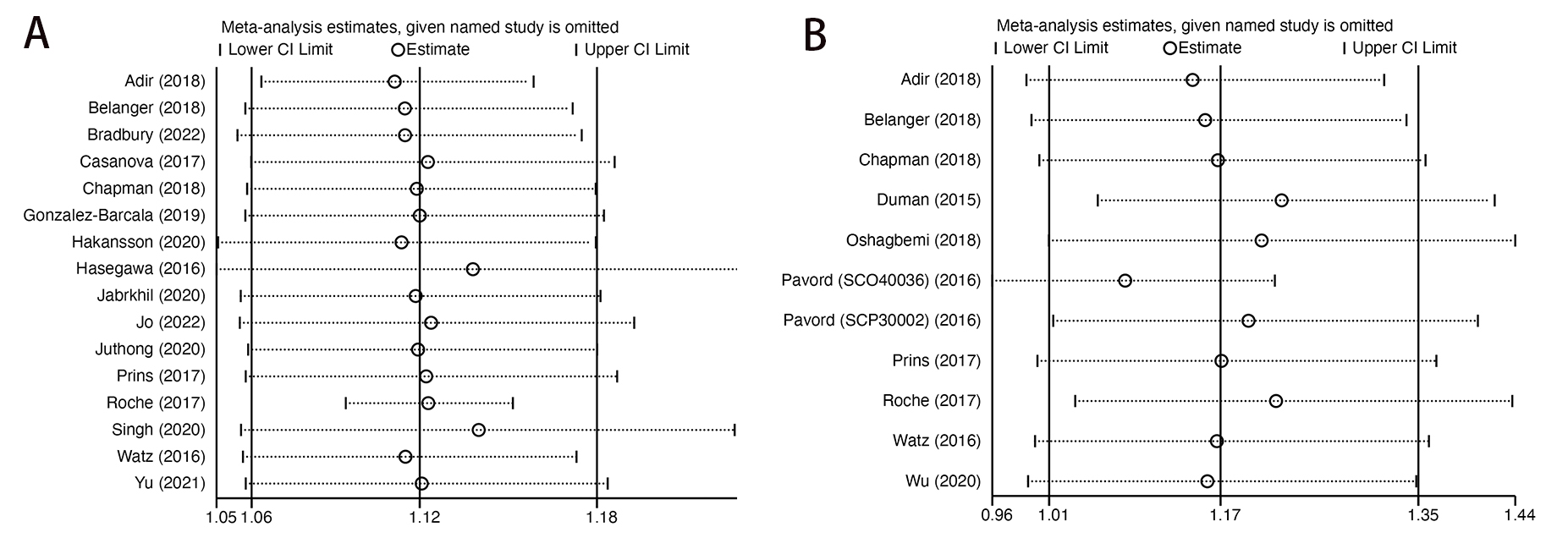
**S4 Fig.** Sensitivity analysis, based on the thresholds of 300 cells/μL (A) and 2% (B). Further exclusion of any single study did not materially alter the pooled RRs.
