# Supplementary material for: High blood eosinophils predict the risk of COPD exacerbation: A systematic review and meta-analysis
Source: PLoS One. 2024 Oct 3;19(10):e0302318. doi: 10.1371/journal.pone.0302318 (PMC11449345; doi:10.1371/journal.pone.0302318)
Supplement: S5 Fig — (A) Funnel plot. (B) Egger’s publication bias plot. (DOCX) [file pone.0302318.s009.docx]

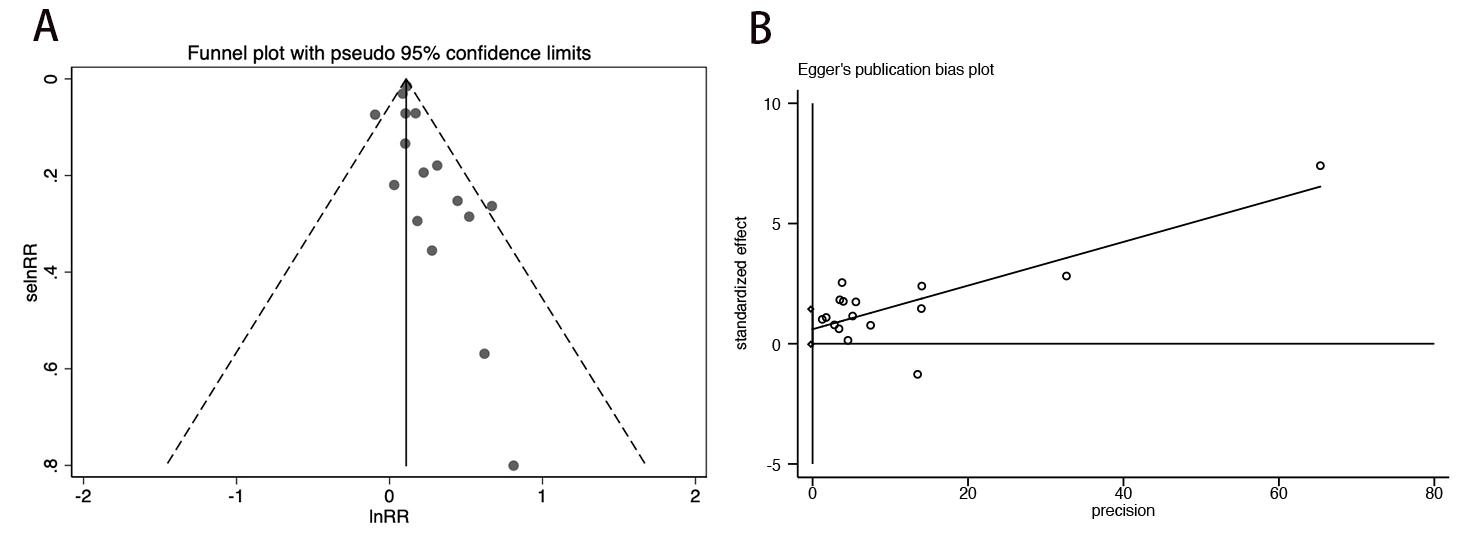
 **S5 Fig.** Publication bias, based on the threshold of 300 cells/μL. (A) Funnel plot. (B) Egger's publication bias plot.
